# Comparison of nutritional screening tools in pediatric patients undergoing hematopoietic stem cell transplantation

**DOI:** 10.3389/fped.2026.1954669

**Published:** 2026-09-24

**Authors:** Lixian Han, Yun Zhuang, CaiQin Zhang, Ying Xiong, Juan Li, Wenmin Zhou, Ping Li, Huifang Wu

**Affiliations:** 1 Department of Hematology, Children's Hospital of Soochow University, Suzhou, China; 2 Department of Clinical Nutrition, Children's Hospital of Soochow University, Suzhou, China

**Keywords:** infection, malnutrition, nutritional risk screening, pediatric allogeneic hematopoietic stem cell transplantation, skeletal muscle mass index

## Abstract

**Background:**

Malnutrition is prevalent in children with hematologic malignancies undergoing allogeneic hematopoietic stem cell transplantation (HSCT) and is associated with elevated post-transplant infection risk. No standardized nutritional screening tool exists for this population. This study aimed to compare the performance of four tools (STRONGkids, PYMS, STAMP, and SCAN) in assessing malnutrition risk, and to explore the association between their risk classification and post-transplant infection.

**Methods:**

A total of 199 children with hematologic malignancies who underwent HSCT were enrolled. According to the Global Leadership Initiative on Malnutrition (GLIM) criteria, pre-transplant malnutrition was defined using a CT-derived skeletal muscle index (SMI) Z-score < −2 as the phenotypic criterion for reduced skeletal muscle mass, combined with the underlying hematologic malignancy as the etiologic criterion. This study compared the agreement between the risk classifications assessed by the four tools (STRONGkids, PYMS, STAMP, and SCAN) and GLIM-defined malnutrition diagnosis, as well as the correlation between these risk classifications and post-transplant infection.

**Results:**

Among the 199 children, 53 (26.6%) were diagnosed with malnutrition. The detection rates of high nutritional risk differed significantly among the four screening tools (PYMS 85.43%, SCAN 73.87%, STAMP 58.79%, STRONGkids 48.24%). In terms of screening performance, STRONGkids had an AUC of 0.801, a specificity of 67.8%, and the best agreement with the GLIM criteria (*κ* = 0.479); PYMS had the highest sensitivity (98.1%) but a specificity of only 19.2%. The 100-day post-transplant infection rate was 48.7%, and the high nutritional risk groups identified by all four tools had significantly higher infection rates (all *P* < 0.001). Among them, STRONGkids had an AUC of 0.743 and a Youden index of 0.487; the AUCs of SCAN, STAMP, and PYMS were 0.735, 0.681, and 0.642, respectively.

**Conclusion:**

STRONGkids showed potential application advantages in identifying malnutrition risk and discriminating post-transplant infection in HSCT children.

## Introduction

Pediatric hematologic malignancies pose a major threat to the health of children and adolescents. Allogeneic hematopoietic stem cell transplantation (HSCT) remains the cornerstone curative approach for high-risk, relapsed leukemia, lymphoma, and other hematological neoplasms, offering substantially prolonged long-term survival (1, 2). However, the compounded effects of high-dose myeloablative conditioning chemotherapy before HSCT, post-transplant immunosuppression, graft-versus-host disease (GVHD), and gastrointestinal mucosal injury frequently trigger anorexia, vomiting, diarrhea, and feeding intolerance, which in turn lead to muscle wasting and aberrant body composition. Consequently, malnutrition has become one of the most prevalent comorbidities in pediatric HSCT recipients (3, 4). Unlike children with solid tumors or those receiving conventional chemotherapy, malnutrition in HSCT recipients is not simply reflected by decreased weight or body mass index (BMI); rather, it is characterized predominantly by skeletal muscle depletion (5). Importantly, skeletal muscle loss directly undermines immune reserves, significantly elevating the risks of post-transplant infection, graft failure, prolonged hospital stay, and even mortality, thereby critically compromising transplant outcomes (6). Therefore, accurate identification of children at high risk of malnutrition before transplantation may facilitate early implementation of individualized nutritional intervention, thereby reducing transplant-related complications and improving survival outcomes in pediatric HSCT recipients.

Currently, the main pediatric nutritional risk screening tools used in clinical practice are the Screening Tool for Risk of Nutritional Status and Growth (STRONGkids), the Pediatric Yorkhill Malnutrition Score (PYMS), the Screening Tool for the Assessment of Malnutrition in Pediatrics (STAMP), and the Nutrition Screening Tool for Childhood Cancer (SCAN), the last of which was specifically developed for pediatric oncology patients. These four tools differ markedly in their assessment dimensions and scoring criteria, and to date, there is no universally accepted screening tool that is most suitable for pediatric HSCT recipients (7). Although several studies have compared these pediatric nutrition risk screening tools, the majority have relied on anthropometric measures such as BMI and mid-upper arm circumference as the reference standard for malnutrition identification (8–10). However, anthropometric parameters merely reflect changes in weight and height and cannot precisely quantify skeletal muscle mass, thereby failing to objectively capture the characteristic muscle depletion seen in HSCT recipients.

The Global Leadership Initiative on Malnutrition (GLIM) diagnostic criteria for malnutrition explicitly state that reduced skeletal muscle mass is one of the phenotypic criteria for malnutrition diagnosis and can be combined with an etiologic criterion (such as disease-related inflammation or a catabolic state) to establish a diagnosis of malnutrition (11, 12). The skeletal muscle index (SMI) Z-score can accurately quantify whole-body skeletal muscle mass via abdominal CT and has been recommended as an objective tool for quantifying muscle mass. It compensates for the limitations of traditional height and weight indicators and is more suitable for assessing the nutritional status of pediatric HSCT recipients (13). However, no study has used CT-derived SMI as an objective quantitative indicator of reduced skeletal muscle mass within the GLIM framework to simultaneously evaluate the screening performance of the above four nutritional risk screening tools in pediatric HSCT recipients.

Existing studies suggest that malnutrition is strongly associated with an increased risk of post-transplant infection. Insufficient muscle reserves impair neutrophil recovery and lymphocyte immune function, thereby heightening susceptibility to bacterial, fungal, and viral infections (14). Notably, the first 100 days post-transplantation represent a high-incidence window for early infection and a critical observation period affecting transplant outcomes. Therefore, early identification of high-risk children through nutritional risk screening may provide a basis for stratified management and has potential value for improving transplant outcomes. However, the correlation between different nutritional screening tools and early infection after HSCT remains unclear, and clinical evidence linking malnutrition risk to post-transplant infection is lacking to support the establishment of a standardized nutritional screening and infection risk monitoring workflow in clinical practice.

In summary, the primary objective of this study was to systematically compare the accuracy of four nutritional screening tools (STRONGkids, PYMS, STAMP, and SCAN) in assessing malnutrition risk in pediatric HSCT recipients, using CT-derived SMI Z-score < −2 as the phenotypic criterion for reduced skeletal muscle mass and the GLIM framework as the diagnostic standard for malnutrition. The secondary objective was to explore the association between each tool and infection events within 100 days after transplantation. The aim was to identify a bedside screening tool suitable for pediatric HSCT recipients that combines good nutritional risk screening performance with potential for infection risk stratification, thereby providing evidence for standardized nutritional risk screening workflows and infection stratified management in pediatric HSCT wards.

## Methods

### Study design and participants

This was a single-center retrospective study. We included children with hematologic malignancies (including acute lymphoblastic leukemia (ALL), acute myeloid leukemia (AML), and lymphoma) who underwent allogeneic HSCT at the Department of Hematology, Children's Hospital of Soochow University, between July 2021 and June 2026. All patients were aged 1 to 16 years. We collected baseline clinical data, abdominal CT images, nutritional screening scores (STRONGkids, PYMS, STAMP, and SCAN), and all medical records related to infections within 100 days post-transplant. Exclusion criteria were as follows: congenital myopathy or severe skeletal deformity precluding accurate measurement of skeletal muscle area, pre-transplant active severe infection or multi-organ failure, autologous or second/multiple transplants, and missing key clinical data.

### Malnutrition assessment

This study strictly followed the GLIM diagnostic framework, according to which the diagnosis of malnutrition requires the simultaneous presence of at least one phenotypic criterion and at least one etiologic criterion (11). In our cohort, the etiologic criterion was the underlying hematologic malignancy (ALL, AML, or lymphoma), and these chronic disease states are often accompanied by systemic inflammation and reduced food intake due to chemotherapy-related gastrointestinal toxicity. The phenotypic criterion was reduced skeletal muscle mass, assessed by an SMI Z-score < −2 measured at the L3 level on CT, as detailed below. Abdominal CT cross-sectional images at the L3 vertebral level were uniformly extracted for each patient before transplantation. Two uniformly trained radiologists independently used 3D Slicer to identify muscle tissue and measure skeletal muscle area using a predefined Hounsfield unit threshold (−29 to +150 HU). Disagreements were resolved through discussion, and if necessary, adjudicated by a third senior radiologist. The SMI was computed as SMI = muscle area (cm^2^)/height^2^ (m^2^). SMI Z-scores were derived from a previously published population-based reference model of L3-SMI in healthy children (15, 16). Based on prior literature and the conventional statistical cutoff of Z-score < −2 (i.e., 2 standard deviations below the reference mean), we defined SMI Z-score < −2 as reduced skeletal muscle mass (17, 18). All CT scans were completed within one week before transplantation, within the same time window as nutritional screening. As part of routine pre-transplant clinical evaluation, the primary purposes were to screen for potential infection, assess primary tumor status, and establish baseline imaging data; therefore, no additional radiation exposure was incurred for research purposes.

### Nutritional risk screening

All patients underwent concurrent assessment with the four scales (STRONGkids, PYMS, STAMP, and SCAN) by the responsible nurse within 1 week before transplantation. The median interval between nutritional screening and CT imaging was 1 day (range: 0–3 days).

STRONGkids is applicable for children aged 1 month to 18 years and consists of seven items: subcutaneous fat/muscle wasting, recent weight loss, nutritional intake and losses, and presence of a high-risk underlying disease. Each item is scored dichotomously, with total scores ranging from 0 to 5. Risk stratification was defined as follows: 0 = low risk, 1–3 = moderate risk, and 4–5 = high risk (19). Because hematologic malignancies are severe consumptive high-risk diseases, all patients in our cohort were categorized as either moderate or high risk, with no patients assigned to the low-risk group.

PYMS consists of four core domains: BMI, weight change over the past 3 months, recent reduction in intake, and the impact of disease on intake. Each domain is scored according to severity, with total scores ranging from 0 to 7. A total score ≥ 2 was used as the cutoff for high nutritional risk (20).

STAMP includes three modules: disease type, dietary intake, and anthropometric measures, each with stratified scoring. Total scores range from 0 to 9, with risk classified as low (0–1), moderate (2–3), or high (≥ 4) (21). In our cohort, all patients were categorized as either moderate or high risk, with no patients assigned to the low-risk group.

SCAN is a specific screening tool developed for pediatric oncology patients. It comprises six items relevant to cancer treatment: tumor risk stratification, treatment intensity, gastrointestinal adverse effects, oral intake over the past week, weight loss over the past month, and clinically evident signs of malnutrition. Each item is scored by severity, with total scores ranging from 0 to 10. A total score ≥ 3 indicates high nutritional risk (22).

### Infection assessment

The occurrence of infection within 100 days after transplantation was recorded, including bacterial, fungal, and viral infections. Any single infection event, regardless of grade, was recorded as positive. Infection categories included bloodstream infection, pulmonary infection, gastrointestinal infection, and skin and soft tissue infection. Infection outcomes were double-blind reviewed by two transplant specialists, and only infection events with both typical infection-related clinical manifestations and at least one piece of objective evidence (microbiological, imaging, or laboratory findings) were included as positive outcomes.

The diagnostic criteria for each infection type were as follows: Bloodstream infection (BSI): at least two separate blood culture samples drawn on the same occasion, with ≥1 bacterial or fungal pathogen detected in at least two different blood culture samples, or ≥1 common commensal organism, accompanied by clinical signs or symptoms of systemic infection. Pulmonary infection: new infiltrates on chest CT, plus at least one of the following: fever (>38.0 °C), cough, sputum production, dyspnea, or positive respiratory pathogen testing (culture, PCR, or antigen detection). Gastrointestinal infection: diarrhea (≥3 loose stools per day for ≥2 days) or vomiting, plus at least one of the following: positive stool culture/PCR for enteric pathogens, or endoscopic/histopathological evidence of infection. Skin and soft tissue infection: local erythema, swelling, warmth, or purulent discharge, with positive culture from skin/swab or blood, or biopsy-confirmed infection.

### Statistical analysis

We used the Kolmogorov–Smirnov test to assess the distribution of continuous variables. Normally distributed data are presented as mean ± standard deviation, while non-normally distributed data as median (interquartile range). Categorical variables are described as numbers and percentages. Comparisons between two groups were performed using the independent-samples *t*-test, Mann–Whitney U test, chi-square test, or Fisher's exact test, as appropriate.

Using GLIM-diagnosed malnutrition as the standard, 2 × 2 contingency tables were constructed to calculate the sensitivity, specificity, positive predictive value, and negative predictive value of each of the four screening tools. ROC curves were plotted and the AUC of each tool was compared. The Kappa test was used to evaluate the agreement between each screening tool and the GLIM reference standard, as well as the pairwise agreement among the four tools. For the secondary exploratory analysis of infection outcomes, the chi-square test or Fisher's exact test was used to analyze the association between the high nutritional risk classification of each scale and the occurrence of infection. For each of the four screening tools, sensitivity, specificity, positive predictive value (PPV), negative predictive value (NPV), and Youden index were calculated, and ROC curves were plotted to compare the AUC values for discriminating infection status. All statistical analyses were performed using SPSS (version 26.0). A two-sided *P* < 0.05 was considered statistically significant.

### Ethical considerations

This study protocol was approved by the Institutional Review Board of the Children's Hospital of Soochow University (approval number: 2026CS133). The procedures complied with the Declaration of Helsinki. After initial screening based on the inclusion and exclusion criteria, eligible children and their parents were informed about the study, and written informed consent was obtained from the individuals legally responsible for the children.

## Results

### Study population

A total of 199 patients who met the inclusion and exclusion criteria were enrolled. The cohort consisted of 101 boys (50.7%) and 98 girls (49.3%), with a mean age of 8.4 years (range, 1–16 years). Age distribution was as follows: 36 patients (18.1%) were aged ≤ 3 years, 37 (18.6%) were aged 4–6 years, 78 (39.2%) were aged 7–12 years, and 48 (24.1%) were aged 13–16 years. The most common underlying disease was ALL, occurring in 108 patients (54.3%), followed by AML (*n* = 44, 22.1%) and lymphoma (*n* = 47, 23.6%).

### Nutritional status and nutritional risk screening results

Based on the GLIM criteria, 53 patients (26.6%) were diagnosed with malnutrition. The four screening tools showed marked differences in risk stratification. STRONGkids classified 103 patients (51.76%) as moderate risk and 96 (48.24%) as high risk; STAMP classified 82 (41.21%) as moderate risk and 117 (58.79%) as high risk; SCAN identified 147 patients (73.87%) as high risk; and PYMS identified the highest proportion of high-risk patients among the four tools, with approximately 170 patients (85.43%).

Further analysis stratified by age (Table 1) showed no statistically significant difference in the prevalence of malnutrition across age groups (*χ*^2^ = 5.186, *P* = 0.159). Similarly, no significant differences were observed in risk classifications by SCAN and STRONGkids across age groups. However, risk classifications by PYMS and STAMP differed significantly by age (*P* < 0.05). Regarding disease type, the prevalence of malnutrition was markedly higher in AML patients than in those with ALL or lymphoma. All four screening tools showed that the AML group had the highest proportion of patients at high nutritional risk (*P* < 0.05), suggesting that the underlying disease type is an important determinant of nutritional risk.

**Table 1:** Nutritional status and risk stratification by age and disease type.

N		GLIM criteria		STRONGkids		PYMS		STAMP		SCAN	
		Normal	Malnutrition	Moderate risk	High risk	Low-moderate risk	High risk	Moderate risk	High risk	Risk-free	High risk
Nutritional risks	199	146 (73.4)	53 (26.6)	103 (51.8)	96 (48.2)	29 (14.6)	170 (85.4)	82 (41.2)	117 (58.8)	52 (26.1)	147 (73.9)
Age											
＜3 years	36	22 (61.1)	14 (38.9)	14 (38.9)	22 (61.1)	3 (8.3)	33 (91.7)	18 (50.0)	18 (50.0)	10 (27.8)	26 (72.2)
4–6 years	37	31 (83.8)	6 (16.2)	23 (62.2)	14 (37.8)	10 (27.0)	27 (73.0)	16 (43.2)	21 (56.8)	11 (29.7)	26 (70.3)
7–12 years	78	59 (75.6)	19 (24.4)	46 (59.0)	32 (41.0)	2 (2.6)	76 (97.4)	23 (29.5)	55 (70.5)	15 (19.2)	63 (80.8)
13–18 years	48	34 (70.8)	14 (29.2)	20 (41.7)	28 (58.3)	14 (29.2)	34 (70.8)	25 (52.1)	23 (47.9)	16 (33.3)	32 (66.7)
x^2^		5.186		7.576		22.983		7.978		3.513	
*P*		0.159		0.056		<0.001		0.046		0.319	
Diagnosis											
ALL	108	86 (79.6)	22 (20.4)	73 (67.6)	35 (32.4)	28 (25.9)	80 (74.1)	49 (45.4)	59 (54.6)	48 (44.4)	60 (55.6)
AML	44	17 (38.6)	27 (61.4)	7 (15.9)	37 (84.1)	1 (2.3)	43 (97.7)	10 (22.7)	34 (77.3)	2 (4.5)	42 (95.5)
Lymphoma	47	43 (91.5)	4 (8.5)	23 (48.9)	24 (51.4)	0 (0)	47 (100)	23 (48.9)	24 (51.1)	2 (4.3)	45 (95.7)
x^2^		37.229		33.642		24.547		8.134		41.038	
*P*		<0.001		<0.001		<0.001		0.017		<0.001	

### Value of the four tools for nutritional risk screening in hospitalized children

The high-risk classifications from all four screening tools showed a significant association with malnutrition diagnosed by the GLIM criteria based on SMI Z-score (all *P* < 0.01). Analysis of sensitivity and specificity showed that the PYMS scale had the highest sensitivity for identifying malnutrition, reaching 98.1%, and the STRONGkids scale had the highest specificity at 67.8%. In addition, STRONGkids had the highest PPV among the four scales (51.0%), and all scales had NPVs exceeding 90%, with PYMS having the highest NPV (96.6%). Furthermore, compared with the other three scales, STRONGkids had the highest agreement with malnutrition (*κ* = 0.479, *P* < 0.001). ROC analysis using malnutrition as the standard showed that STRONGkids had the highest AUC (AUC = 0.801, 95% CI 0.737–0.866, *P* < 0.001) (Table 2).

**Table 2:** Diagnostic performance of four nutritional screening tools.

Screening tools	X^2^	*P*	Sensitivity (%)	Specificity (%)	PPV	NPV	Kappa	*P*
STRONGkids	47.198	<0.001	92.5 (49/53)	67.8 (99/146)	51.0 (49/96)	96.1 (99/103)	0.479	<0.001
PYMS	32.283	<0.001	98.1 (52/53)	19.2 (28/146)	30.6 (52/170)	96.6 (28/29)	0.102	0.002
STAMP	26.809	<0.001	84.9 (45/53)	50.7 (74/146)	38.5 (45/117)	90.2 (74/82)	0.257	<0.001
SCAN	56.796	<0.001	94.3 (0/53)	33.5 (49/146)	34.0 (50/147)	94.2 (49/52)	0.178	<0.001

Pairwise agreement analysis among the four tools showed that the overall agreement rate ranged from 31.7% (STRONGkids vs. STAMP) to 73.9% (PYMS vs. SCAN), with Kappa values ranging from 0.131 to 0.651. The agreement between PYMS and SCAN was the highest (*κ* = 0.651), whereas that between STRONGkids and STAMP was the lowest (*κ* = 0.131, *P* = 0.059) (Table 3).

**Table 3:** Pairwise agreement among the four nutritional risk screening tools.

Screening tools	Low + moderate	High risk	kappa	*p*值
Agreement = 48.2%	STRONGkids			
PYMS				
Low + moderate	29 (28.2%)	0 (0%)	0.274	0.000
High risk	74 (71.8%)	96 (100%)		
Agreement = 31.7%	STRONGkids			
STAMP				
Low + moderate	49 (47.6%)	33 (34.4%)	0.131	0.059
High risk	54 (52.4%)	63 (65.6%)		
Agreement = 47.7%	STRONGkids			
SCAN				
Low + moderate	51 (49.5%)	1 (1.0%)	0.476	0.000
High risk	52 (50.5%)	95 (99.0)		
Agreement = 57.3%	PYMS			
STAMP				
Low + moderate	26 (89.7%)	56 (32.9%)	0.323	0.000
High risk	3 (10.3%)	114 (67.1%)		
Agreement = 73.9%	PYMS			
SCAN				
Low + moderate	29 (100%)	23 (13.5%)	0.651	0.000
High risk	0 (0%)	147 (86.5%)		
Agreement =52.8%	STAMP			
SCAN				
Low + moderate	40 (48.8%)	12 (10.3%)	0.408	0.000
High risk	42 (51.2%)	105 (89.7%)		

### Association between nutritional risk classification and post-transplant infection

Among the 199 children, 97 (48.7%) developed infection within 100 days after transplantation. The infection rate in children diagnosed with malnutrition according to the GLIM criteria was significantly higher than that in children without malnutrition (75.4% vs. 39.0%, *P* < 0.001). When stratified by the four screening tools, the infection rates in the high-risk groups identified by all four tools were significantly higher than those in the low-to-moderate-risk groups (all *P* < 0.001). Among the high-risk groups, the highest infection rate was observed for STRONGkids (73.9%), followed by SCAN (64.6%), STAMP (64.1%), and PYMS (57.0%). Notably, no infection cases occurred in the low-to-moderate-risk group of PYMS (0/29) (Table 4).

**Table 4:** 100-day post-transplant infection occurrence by nutritional risk level.

Screening tools	Group	*N*	Infection	x^2^	*P*
		199	97 (48.7)		
GLIM	Normal	146	57 (39.0)	20.656	<0.001
	Malnutrition	53	40 (75.4)		
STRONGkids	Moderate risk	103	26 (25.2)	47.198	<0.001
	High risk	96	71 (73.9)		
PYMS	Low-moderate risk	29	0 (0.0)	32.283	<0.001
	High risk	170	97 (57.0)		
STAMP	Moderate risk	82	22 (26.8)	26.809	<0.001
	High risk	117	75 (64.1)		
SCAN	Risk-free	52	2 (3.8)	56.796	<0.001
	High risk	147	95 (64.6)		

ROC analysis for discriminating infection status showed that the AUC was 0.743 (95% CI 0.673–0.814, *P* < 0.001) for STRONGkids, 0.735 (95% CI 0.664–0.805, *P* < 0.001) for SCAN, 0.681 (95% CI 0.606–0.756, *P* < 0.001) for STAMP, and 0.642 (95% CI 0.565–0.719, *P* = 0.001) for PYMS. STRONGkids had the highest Youden index (0.487), with a sensitivity of 73.2% and a specificity of 75.5% (Table 5).

**Table 5:** Predictive performance of four nutritional screening tools for 100-day post-transplant infection.

Screening tools	AUC (95% CI)	*P*	Sensitivity (%)	Specificity (%)	PPV	NPV	Youden's index
STRONGkids	0.743 (0.673–0.814)	<0.001	73.2 (71/97)	75.5 (77/102)	74.0 (71/96)	74.8 (77/103)	0.487
PYMS	0.642 (0.565–0.719)	0.001	100 (97/97)	0.284 (29/102)	57.1 (97/170)	100 (29/29)	0.284
STAMP	0.681 (0.606–0.756)	<0.001	77.3 (75/97)	58.8 (60/102)	64.1 (75/117)	73.2 (60/82)	0.361
SCAN	0.735 (0.664–0.805)	<0.001	97.9 (95/97)	49.0 (50/102)	64.6 (95/147)	96.2 (50/52)	0.469

## Discussion

Malnutrition remains an important clinical issue for children with hematologic malignancies undergoing HSCT. The high aggressiveness of the underlying disease, along with treatment-related injuries before transplantation, frequently leads to feeding difficulties and substantial skeletal muscle loss, which in turn undermines immune reserves and increases the risk of post-transplant infection. Previous studies have suggested that pre-transplant muscle wasting in pediatric hematologic malignancy patients may be associated with higher non-relapse mortality (NRM), worse overall survival (OS), and worse event-free survival (EFS) (6). Therefore, how to select a nutritional screening tool suitable for pediatric HSCT recipients to rapidly and accurately assess malnutrition risk has become a key clinical issue that urgently needs to be addressed.

The reported prevalence of malnutrition in pediatric oncology populations varies widely, ranging from 6.1% to 88.4%, and diagnostic criteria are the primary factor contributing to heterogeneity across studies (23). In this study, GLIM was used as the diagnostic standard for malnutrition, with CT-derived L3 skeletal muscle index (SMI) Z-score < −2 as an objective indicator of reduced skeletal muscle mass. The pre-transplant malnutrition rate among the 199 children was 26.6%, which is much higher than the 6.8% reported by Hardenson Rodríguez González et al. based on anthropometric measures in children with hematologic and lymphatic malignancies (24). This difference may be due to the use of a CT-based skeletal muscle index in this study to directly quantify skeletal muscle mass, which provides a more objective assessment of changes in skeletal muscle mass compared with routine anthropometry. Notably, the malnutrition rate in children with AML in this study was significantly higher than in those with ALL or lymphoma. Myeloid malignancies are accompanied by persistent systemic inflammation, and combined with the gastrointestinal toxicity of intensive chemotherapy, this leads to a much greater degree of muscle loss than in children with lymphoid malignancies (25). This stratification pattern is consistent with findings from previous pediatric oncology cohort studies, further supporting that skeletal muscle-centered GLIM criteria may have higher specificity in identifying malnutrition in children (26).

To our knowledge, this study is the first to use CT-derived SMI Z-score as a quantitative indicator of reduced skeletal muscle mass within the GLIM framework and as a reference standard for malnutrition, comparing the identifying performance of four commonly used pediatric nutritional risk screening scales in the HSCT population. Marked differences were observed in the proportion of high nutritional risk detected by the four pediatric nutritional screening tools, with STRONGkids showing the highest agreement with the GLIM reference standard. Similarly, a study by Hong Chen et al. found that compared with PYMS, the STRONGkids tool showed higher identifying ability in distinguishing WHO-defined malnutrition in hospitalized children (AUC: 0.812 vs. 0.738) (27). However, in the study by Buket Meral et al., compared with STRONGkids and STAMP, PYMS had the best specificity (96.4%) and sensitivity (79.7%) in identifying patients at risk of malnutrition determined by SCAN in children with hematologic malignancies (28). This difference may be due to differences in the assessment dimensions and malnutrition standards across the tools, rather than simply reflecting differences in the nutritional status of the populations. The STRONGkids scale contains direct clinical evaluation items for muscle and fat depletion and is the only screening tool that incorporates clinical assessment of muscle depletion into its items. This feature may bring STRONGkids closer to the skeletal muscle-based GLIM reference standard. This may partially explain why the STRONGkids scale showed the highest agreement with the malnutrition diagnostic standard in this study population. Although the sensitivity of PYMS and SCAN was prominent, reaching 98.1% and 94.3%, respectively, their specificity was relatively low. This may be because the SCAN scale contains items related to chemotherapy intensity and gastrointestinal symptoms, which tend to score higher in HSCT children, while PYMS mainly relies on recent weight changes and short-term appetite status, which also tends to elevate scores. Pairwise agreement analysis showed that PYMS and SCAN had the highest agreement (*κ*= 0.651), suggesting a similar tendency toward broadly identifying high risk, whereas the agreement between STRONGkids and STAMP was nearly random (*κ*= 0.131), reflecting fundamental differences in their assessment dimensions. These differences reflect the different assessment dimensions emphasized in the design of each tool, rather than a defect in any single tool itself. All four scales had relatively high NPVs. Children classified as low risk by any tool essentially had no malnutrition risk, suggesting that these tools have potential triage value in the initial screening stage and may help reduce radiation exposure and healthcare costs associated with CT imaging assessment.

Nutritional status serves as a critical foundation for immune cell proliferation and gastrointestinal mucosal repair. Inadequate nutrition directly delays immune reconstitution after myeloablative conditioning and increases the risk of infection, which is of paramount importance for the prognosis of HSCT recipients (29). Therefore, this study associated the four screening tools with the prognostic outcome of infection within 100 days after transplantation, initially exploring the potential value of nutritional risk screening for post-transplant infection. Previous studies have confirmed that reduced skeletal muscle mass is an independent prognostic factor for post-transplant infection in children with hematologic malignancies, but studies on the correlation between nutritional screening scales and infection are relatively few. Triarico et al. studied 126 pediatric oncology patients and found that those identified as high-risk by STRONGkids had a significantly increased infection risk, with high nutritional risk at diagnosis being an independent predictor of infection on multivariate analysis (30). Our study showed that children at high risk by STRONGkids had the highest nosocomial infection rate (73.9%), and in discriminating post-transplant infection, the AUC value (0.743) and Youden index (0.487) were higher than those of the other three tools. This corresponds to the results of Hong Chen et al., who found that the incidence of nosocomial infection in STRONGkids high-risk children (66.27%) was higher than that in PYMS high-risk children (62.50%) (27). It is worth noting that the good performance of STRONGkids in discriminating post-transplant infection does not equate to a causal relationship from malnutrition identification to infection prediction. This may be related to the muscle depletion assessment item included in STRONGkids, which captures nutritional status information associated with post-transplant immune recovery. However, this hypothesis still needs to be verified in future prospective studies. From a clinical practice perspective, the association between nutritional risk screening and infection also has potential application value. Currently, simple tools available in routine clinical practice for early identification of children at high risk of infection remain limited. After nutritional screening tools initially determine infection risk stratification before transplantation, clinical teams can perform closer peri-transplant monitoring for high-risk children, including more frequent vital sign observation, dynamic monitoring of infection-related laboratory indicators, and early evaluation of prophylactic anti-infection measures. Its actual value can be verified through future prospective interventional studies.

## Strengths and limitations

This study has several strengths. First, although some studies have investigated the performance of pediatric nutritional risk screening tools, comparing four widely used tools (PYMS, STAMP, STRONGkids, and SCAN) using GLIM criteria based on skeletal muscle quantification is an important strength of this study, because it overcomes the inherent limitations of traditional anthropometry and can more objectively reflect the nutritional status of pediatric HSCT recipients. Second, our study associated the four screening tools with a key short-term prognostic outcome, infection within 100 days after transplantation, providing preliminary clues for the potential value of nutritional risk screening in the early warning of early post-transplant complications.

Several limitations should be acknowledged. First, this was a single-center retrospective study with a relatively limited sample size, and the external generalizability of our findings needs to be confirmed by larger, multicenter prospective studies. Second, the CT-derived SMI Z-score reference values at the L3 level were based on pediatric data from a single country. Whether population-specific reference standards from different regions would lead to different classifications warrants further investigation. Third, the infection analysis in this study was exploratory and did not adjust for potential confounders. Future large-sample prospective studies with multivariable adjustment are needed to further verify the association between nutritional risk classification and post-transplant infection. In addition, this design can only suggest an association, and whether screening-guided intensified intervention can improve infection outcomes still requires further research for verification. Fourth, there is conceptual overlap between STRONGkids and the reference standard of this study in the dimension of “muscle assessment,” and its performance advantage should be interpreted with caution. In addition, this study did not perform a formal statistical comparison of AUCs. In the infection analysis, the AUC difference between STRONGkids and SCAN was small, and this should not be overinterpreted as STRONGkids having clinical superiority.

## Conclusion

Selecting an appropriate nutritional screening tool is crucial for nutritional care and prognostic management in children with hematologic malignancies. This study compared four commonly used nutritional risk screening tools, and the results showed that STRONGkids had the best intrinsic agreement with the GLIM malnutrition reference standard based on skeletal muscle and demonstrated potential application advantages in discriminating post-transplant infection. This study provides a reference basis for identifying patients who may require intensified nutritional monitoring, targeted nutritional support, and infection prevention. However, its clinical utility still needs to be further verified in prospective studies.

## Data Availability

The original contributions presented in the study are included in the article/Supplementary Material, further inquiries can be directed to the corresponding author.
